# Factors Affecting Health Care Utilization in Tehran

**DOI:** 10.5539/gjhs.v7n6p240

**Published:** 2015-04-16

**Authors:** Soraya Nouraei Motlagh, Asma Sabermahani, Mohammad Hadian, Mohsen Asadi Lari, Mohamad Reza Vaez Mahdavi, Hassan Abolghasem Gorji

**Affiliations:** 1 Department of Health Economics, School of Health Management and Information Sciences, Iran University of Medical Sciences, Tehran, Iran; 2 Research Center for Health Services Management, Institute for Futures Studies in Health, Kerman University of Medical Sciences, Kerman, Iran; 3 Department of Epidemiology, School of Public Health and Oncopathology Research Centre, Iran University of Medical Sciences, Tehran, Iran; 4 Department of Physiology, Faculty of Medicine, Shahed University, Tehran, Iran; 5 Department of Health Services Management, School of Health Management and Information Sciences, Iran University of Medical Sciences, Tehran, Iran

**Keywords:** health services utilization, access to care, Urban-HEART, Tehran

## Abstract

**Introduction:**

Successful health system planning and management is dependent on well informed decisions, so having complete knowledge about medical services’ utilization is essential for resource allocation and health plans. The main goal of this study is identification of factors effecting inpatient and outpatient services utilization in public and private sectors.

**Methods:**

This study encompasses all regions of Tehran in 2011 and uses Urban HEART questionnaires. This population-based survey included 34700 households with 118000 individuals in Tehran. For determining the most important factors affected on health services consumption, logit model was applied.

**Results:**

Regarding to the finding, the most important factors affected on utilization were age, income level and deciles, job status, household dimension and insurance coverage. The main point was the negative relationship between health care utilization and education but it had a positive relationship with private health care utilization. Moreover suffering from chronic disease was the most important variable in health care utilization.

**Conclusions:**

According to the mentioned results and the fact that access has effect on health services utilization, policy makers should try to eliminate financial access barriers of households and individuals. This may be done with identification of households with more than 65 or smaller than 5 years old, people in low income deciles or with chronic illness. According to age effect on health services usage and aging population of Iran, results of this study show more importance of attention to aged population needs in future years.

## 1. Introduction

Successful health system planning and management is dependent on well informed decisions, so having complete knowledge about medical services’ utilization is essential for resource allocation and health plans. (Baker & Liu, 2006)

Access to care is the first essential condition for utilization of services. Access and utilization are related concepts in health sector and access has a fundamental role in use of medical care services. Access to services is complicated terms with various definitions which may change over the time depend on its use. Access to qualified health care services and diagnostic tools is an important part of strategies designed to achieve the Millennium Development Goals and equal access of different social and economic groups to health services is one of the main goals of health policies (Van der Heyden, Demarest, Tafforeau & Van Oyen, 2003). According to public opinion, inequality in the use of medical care services is unjust and unfair (Zyaambo, Siziya, & Fylkesnes, 2012), and health systems should guarantee equal access to medical care according to need (Van der Heyden et al., 2003). Just a service is fair that access to it is based on need, regardless of ability to pay (Palència et al., 2013). Social and economic disparities in utilization of health services lead to increased burden of diseases and resonance social inequalities in health and generate adverse social and economic effects (Dahlgren & Whitehead, 2007), so healthcare researchers and policy makers have been focused on reducing the gap in health disparities in vulnerable populations recently (Song et al., 2010).

There are several models for describing the medical care utilization. Andersen 1995 described the basic model of medical care usage in the form of a behavioral model. In this model people utilization of services is considered as a function of their ability to use, utilization barriers and their need to health care services. Moreover some people have much tendency to use health care services than others that this tendency is predictable by attention to some characteristics. People with these characteristics are more probably to consume health care services due to their much need. These characteristics include demographic, social and motivational characteristics. In addition to predictive characteristics, there should be suitable utilization condition for people to use services. These conditions include Income, health insurance, services availability and people ability to use. Utilization of health services will not occur without these conditions (Andersen & Newman, 1973).

Researches about health care utilization help us to know facilitating factors of access to medical services and appropriate volume and quality of provided health care services (Shin, Song, Kim & Probst, 2005).

After Iran revolution, although we have reached to increase in health care access due to universal insurance coverage and improve in health care indicators, intriguing changes haven’t been occurred in decreasing inequality and differences in health care utilization exist between different groups and provinces (Hassanzadeh, Mohammadbeigi, Eshrati, Rezaianzadeh & Rajaeefard, 2013). Therefore this study was done with aim of identification of factors effecting inpatient and outpatient services utilization in public and private sectors and encompasses all regions of Tehran in 2011 and uses Urban HEART questioners.

## 2. Methodology

For determining utilization we use Urban Health Equity Assessment and Response Tool (Urban HEART) that is recently developed by the WHO Centre for Health Development located in Kobe, Japan (WKC) was improved and employed in Tehran in two stages (2008 and 2011) in 22 regions and 386 districts respectively and gathered valuable data about health conditions in Tehran urban settings (Asadi-Lari & Vaez-Mahdavi, 2011). This data gathering provided suitable condition for wide researches in various aspects in Tehran. One of these researches was about health care services utilization in both private and public sectors. So, researchers using Logit econometric model, have done this research to prepare usable information for planners and policy makers and illustrate actual access to health care services.

For data gathering in 22 municipality regions and 368 districts of Tehran, multi stage (stratify, systematic cluster and systematic) sampling was applied. Sample size 1535 for regions was determined on the base of variables with 10 percent or more prevalence, considering 95 percent confidence and 0.015 errors. Then sample size increased to 1600 households For Facilitating sample allocation with attention to age and sex table of questioners. So in each region, 200 city blocks and in each block, 8 households were selected for the study with systematic sampling. On districts level, sampling method was proportional to size. Comprehensive map of Tehran 2011 was the sample frame of this project. For districts sampling, each district map was put on the coordinate axes and sample blocks were selected with dimensional systematic sampling. Then blocks homes were marked and homes were selected through linear systematic manner. Generally, this population-based survey included 34700 households (with 118000 individuals) in 22 region of Tehran in 2011. There were three kinds of questioner with 21 parts in this survey which 14 first parts (include services utilization questioner) were completed for all 8 selected households in each block.

Some data that was used in this study were information about age, sex, income, education level, chronic diseases of household members, under 5 and over 65 years old people in the household (because of more need to health care), health insurance, and household size and health expenditures.

Data was analyzed with E-views6 software after gathering and justifying in Excel software in several Logit models for inpatients, outpatients, private and public services. Whenever dependent variable in research is binary, logit model is applied. In this research, Dependent variable is health services utilization. Value of this variable for utilization of medical services is 1, and for not utilization is zero. Firstly in this model, probability of health services use is found, afterwards, the marginal effect for each independent variable is obtained. The marginal effect of each explanatory variable indicates the amount of change in probability of the Occurrence of explained variable because of a unit increase in each explanatory variable. Each model was specified and Hosmer-Lemeshow test was used for checking goodness of fit and Davidson and MacKinnon test for heteroskedasticity of models.

## 3. Results

The research results show that in 2011, generally Tehran’s people used 40.7 percent outpatient and 18.69 percent inpatient health services. Household head characteristics and utilization rate of services groups in each region are presented in Tables 1 and 2. Households represented various reasons for no utilization of health services that are shown in Table 2. In some cases two or more reasons are represented, that are shown in the table respectively.

**Table 1:** Household Head Characteristics

Variable	Categories	Percent
Education	Primary	23.48
	Secondary	56.35
	Academic	20.17
Sex	Female	11
	Male	89
Group Income	Group 1 (Lowest group)	22.31
	Group 2	20.76
	Group 3	19.55
	Group 4	18.94
	Group 5 (Highest group)	18.44
Age Range	Under 30 years	5.50
	Between 30-60 years	69.27
	Upper 60 years	25.23

**Table 2:** Health services utilization percent in Tehran various regions

Region	Total inpatient utilization	Private inpatient utilization	Total outpatientutilization	Most important reasons of no inpatient services utilization	Most important reasons of no outpatient services utilization
1	16.67	55	45	No response	Existence of drug at home and services expensiveness
2	18.42	44.3	39.41	No existence of the desired specialist at the facility	Decision for future refer and existence of drug at home
3	14.8	58.6	35.2	No response	Having no time and distance of the facility
4	24	25	42	No response	No response
5	16.75	32.3	39.55	Having no money and not acceptance in the facility and services expensiveness	Existence of drug at home and self-treatment
6	18.62	51.3	27.22	No turn reaching and no existence of the desired specialist at the facility	Existence of drug at home
7	22.68	28.9	32.87	Having no money and not acceptance in the facility and services expensiveness	Having no money and having no time and existence of drug at home
8	23.37	40.5	40.83	No response	All reasons
9	18.85	24.1	42.91	No turn reaching	Existence of drug at home and services expensiveness
10	18.06	25.5	43.08	Services expensiveness and having no money and no existence of the desired specialist at the facility	Problem disappearance
11	18.36	27.1	43.85	Services expensiveness and having no money and no existence of the desired specialist at the facility	Existence of drug at home and problem disappearance
12	16.02	22.9	39.64	Having no money and services expensiveness and having no contract of facilities with patient insurance	Having no money and existence of drug at home
13	16.76	30.1	33.85	No turn reaching	Existence of drug at home and having no money and services expensiveness
14	20.14	22.2	40	No existence of the desired specialist at the facility and having no money	Existence of drug at home and having no money
15	19.17	16.3	41.79	No existence of the desired specialist at the facility and having no money	Existence of drug at home and having no money
16	15.94	17.4	46.96	Having no money and services expensiveness	Existence of drug at home and services expensiveness
17	13.08	16	33.27	services expensiveness	Having no money
18	18.94	12	43.66	having no contract of facilities with patient insurance	Having no money and services expensiveness
19	24.67	22.76	43.98	No existence of the desired specialist at the facility and having no money and having no contract of facilities with patient insurance	Existence of drug at home and having no money
20	20.31	17.4	39.5	Having no money	Existence of drug at home
21	19.77	33.7	46.71	No existence of the desired specialist at the facility	Problem disappearance
22	18.66	35.07	42	All reasons	Existence of drug at home and having no money
Total	18.69		40.07	No turn reaching and having no money	Existence of drug at home and self-treatment and problem disappearance and having no money

Binary dependent variable in the econometric model, which its results are presented in Table 3, is one for health services utilized households and individuals and zero for not utilized ones. According to the model, households and individuals with chronic patient, over 65 and fewer than 5 years old members and which are located between upper income groups are more probably to consume health services. On the contrary women, more educated people and individuals that live in leased houses are less likely to use these services.

**Table 3:** Estimation results of inpatient services Logit model

Independent variable	Individual Level		Household Level	
	
	Coefficients *(Z statistic)*	Marginal effect	Coefficients *(Z statistic)*	Marginal effect
constant	-2.74 (-20.4)	-	-2.003 (-37.77)	-
education	-0.14 (-3.42)	-0.021	-0.15 (-7.15)	-0.025
Third group of education	-0.34 (-6.39)	-0.049	-0.24 (-9.76)	-0.04
Age	0.003 (1.7)	0.0004	0.003 (6.34)	0.0005
Up 65	0.30 (7.96)	0.042	-	-
Under 5	0.62 (14.79)	0.09	-	-
Employed members number	0.0108 *(0.48)	-	0.027(2.65)	0.0043
insurance(uninsured=1 and insured=2)	0.176 (4.25)	0.0255	0.26 (1.85)	0.0042
Household size	0.0022 *(0.16)	-	0.052(7.71)	0.0083
Income rank	0.24 (19.54)	0.034	0.22 (34.26)	0.034
sex(male=1 and female=2)	-0.074 (-1.66)	-0.011	-0.018 (-1.11)	-0.003
Chronic patient	0.69 (19.26)	0.1	0.73 (42.9)	0.12
Housing tenure (homeowners=1 and renting household=2)	-0.1 (-2.6)	-0.0148	-0.09 (-4.62)	-0.0148

Hosmer-Lemeshow test was used for evaluation of estimated model which its value was 22.1 so models predicted results are satisfactory. Mackinnon test demonstrated not existence of heteroskedasticity and estimated model accepted as a suitable model.

In Table 4 which is about outpatient model results, all variables except age are significant. According results, in outpatient groups, members with chronic disease, upper income groups and insured people, are more probably to use outpatient health care services. Moreover households with more than 65 or less than 5 years old members use more outpatient services too. Outpatient services utilization decrease with increasing in education level of households and individuals, employed members or household size. Moreover living in rental houses lead to decrease in outpatient health care utilization.

**Table 4:** Estimation results of outpatient services Logit model

Independent variable	Individual Level		Household Level	
	
	Coefficients *(Z statistic)*	Marginal Effect	Coefficients *(Z statistic)*	Marginal Effect
constant	-1.02 (-9.93)	-0.9641	-0.86 (-18.74)	-
education	-0.089 (-2.63)	-0.02	-0.089 (-4.56)	-0.019
Third group of education	-0.288 (-6.77)	-0.066	-0.19 (-9.12)	-0.045
Age	-0.001 *(0.74)	-0.0002	-0.0003 *(0.8)	-0.0001
Up 65	0.071 (2.25)	0.013	-	-
Under 5	0.11 (3.25)	0.025	-	-
Employed members number	-0.058 (-3.19)	-0.0147	-0.056 (-6.46)	-0.013
insurance(uninsured=1 and insured=2)	0.087 (2.77)	0.02	0.037 (2.64)	0.009
Household size	0.037 (3.3)	0.0085	0.034 (6.06)	0.0081
Income rank	0.16 (16.73)	0.037	0.16 (29.81)	0.036
sex(male=1 and female=2)	0.028 *(0.64)	0.0065	0.024(1.94)	0.006
Chronic patient	0.41 (14.38)	0.095	0.42 (29.15)	0.01
Housing tenure (homeowners=1 and renting household=2)	-0.01 (-3.6)	-0.023	-0.12 (-7.59)	-0.028

About outpatient services unlike inpatient services women are more likely to use services. Hosmer-Lemeshow test result is 10.48 for this model and Mackinnon test show not heteroskedasticity of the model.

Table 5 illustrates results of inpatient health services utilization in private sector versus public sector. As results show all variables except, number of more than 65 and bellow 5 years old and employed members are significant in the model. According to the model, private inpatient services utilization will increase with increase in education level and mean age of households and individuals. Also households and individuals in upper income groups are more likely to use private inpatient services and increase in household size, living in rental houses and having health insurance, decrease utilization of private inpatient services. Hosmer-Lemeshow value is 3.91 and model has no heteroskedasticity too.

**Table 5:** Estimation results of private versus public services Logit model

Independent variable	Individual Level		Household Level	
	
	Coefficients *(Z statistic)*	Marginal effect	Coefficients *(Z statistic)*	Marginal effect
constant	-2.61 (-9.05)	-	-1.61 (-9.05)	-
education	0.53 (5.81)	0.086	0.16 (3.37)	0.027
Third group of education	1.3 (11.87)	0.24	0.68 (12.44)	0.12
Age	0.015 (4.06)	0.003	0.006 (5.89)	0.001
Up 65	0.07 *(0.97)	0.013	-	-
Under 5	0.0134 *(0.39)	0.006	-	-
Employed members number	0.012 *(0.27)	0.002	0.057(2.63)	0.01
insurance (uninsured=1 and insured=2)	-0.28 (-3.26)	-0.05	-0.047 (-1.71)	-0.009
Household size	-0.13 (-4.96)	-0.023	-0.13 (-8.9)	-0.023
Income rank	0.269 (10.25)	0.047	0.29 (20.08)	0.052
sex(male=1 and female=2)	-0.88 (-8.46)	-0.16	-0.075 (-2.16)	-0.013
Chronic patient	-0.13 (-1.3)	-0.024	-0.16 (-4.28)	-0.028
Housing tenure (homeowners=1 and renting household=2)	-0.49 (-5.88)	-0.078	-0.40 (-9.25)	-0.073

## 4. Discussion

Review of previous studies indicates that health care utilization is associated with other factors such as household socioeconomic status in addition to need to health care. Although related previous studies in Iran were applied in smaller or in different settings but have similar results about factors affecting health care utilization. Generally in health care utilization studies, that each of them assessment different group of factors, income, education, employment, insurance and household size were recognized as effecting factors and in our study these effects was proved. In both inpatient and outpatient groups, having members with more than 65 and less than 5 years old or chronic disease lead to increase in health care utilization and obvious reason of this is more health care needs of these groups. In inpatient and outpatient groups, members with chronic disease is the most affecting factor in the estimated models. Poorreza (Poorreza, Khabiry, & Arab, 2008) reported in his study that over 65 years old people and patient people use more health services. Also, two studies in Brazil and Canada showed that health care utilization among the elderly who have chronic disease and movement disorder is more which is consistent with this study result (Thumé, Facchini, Wyshak & Campbell, 2011; Vingilis, Wade & Seeley, 2007).

Insurance coverage has positive effect on inpatient and outpatient health care utilization and like previous studies (Vingilis, et al., 2007) health insured people use more health services. Also, Wagstaff and Pradhan found health insurance plan in Vietnam lead to increase in outpatient and inpatient services utilization in pediatrics and adults (Wagstaff & Pradhan, 2005). Moreover, Trujillo et al. showed this plan has increasing effect on health care utilization in poor and uninsured people in Colombia (Trujillo, Portillo, & Vernon, 2005). Harmon and Nolan noticed the probability of using inpatient services among insured people is 3 percent more than uninsured (Harmon & Nolan, 2001). This relationship has been confirmed in other studies that all approve this study results (Kiil, 2012; Wagstaff, Lindelow, Jun, Ling & Juncheng, 2009).

Income and income group is one of the most effective determinants on health care utilization and has positive effect on usage of inpatient and outpatient services. Positive effect of household’s income was proved in Uddin study too (Uddin & Mazur, 2014).

In outpatient services group results show that age has no effect on utilization but it is effective about inpatient group. This may be because of increase of inpatient households and individuals’ needs with average age increasing.

Sex were significant in both groups but with different sign. Women used less inpatient care and this may be related to weaker socio-economic condition of them. As these groups used more outpatient services, one may conclude that women need to more inpatient services because of their bad conditions but some problems like having no protection for other household members during hospitalization don’t allow them to use services. Greater use of outpatient services in these groups has been confirmed in different studies (Gerritsen et al., 2006; Mendoza-Sassi & Béria, 2003).

Housing tenure has negative relation with health services utilization in both inpatient and outpatient groups. People with rental homes use fewer services in both kinds and this may be related with weak socio-economic condition.

Household size had positive relation with utilization of health services that is because of each member need and quality of life decreasing and diseases increasing.

Education level had negative effect on inpatient and outpatient utilization so that more educated people used fewer services. The reason may be because of positive relation between health and education level. Moreover this kind of households and individuals use more private services and one may conclude these people’s need is less and they use private services in the need time, this result is similar to some studies done in this field (Karkee & Kadariya, 2013; Pappa & Niakas, 2006). Also, this is coincident with some other researches like Chernew (Chernew, Scanlon, & Hayward, 1998) and Hodgkin (1996) about more importance of quality for educated people. Also, Amaghionyeodiwe (2008) found lower socio-economic people use general physician services and upper ones use more dentists, physiotherapists and specialist physician services.

Number of employed members in a household had positive influence on inpatient utilization but this relation is not significant in the model that may be justified with nature of inpatient services that are unavoidable. This effect in outpatient group was negative and significant that is confirmation of inpatient result case, because these services are not essential as inpatient ones. Some other studies showed that unemployed people use more health services too (Hassanzadeh et al., 2013).

More busy people may use fewer services in this kind. Insignificant positive coefficient of this variable in private and public model shows that households with more employed members in need time use more private services.

In private and public logit model it was shown that there is a positive relation between education, income and age with private inpatient services utilization. In case of education it is because of better welfare level of them and more importance of quality for such people. In the case of income the result is acceptable because of direct relation of income and private services. Positive effect of income on utilization of private services was indicated in Habtom et al.’s study (Habtom & Ruys, 2007). In the case of age variable, it may be reasonable because of better socio-economic situation of older people.

Sex was effective on utilization of private services. Women use less private services and the reason may be worse socio-economic level of them. This conclusion may be true for size of household effect too.

Households who had members with chronic disease, used less private services and this is because of nature of these diseases and need to long term care and higher costs that lead to more use of public services than private one.

Insurance coverage had negative effect on private services usage. One may relate this with more popularity of Social Security insurance and its specific hospitals in Iran that lead to more usage of these hospitals for covered patients. This point leads to negative effect of having insurance on private services utilization in the model. Also this result is due to the fact which around 50% of overall medical care expense in Iran is paid by the individuals and households in the form of direct payment and also private services are not covered by basic insurance. (Manenti, 2011)

Finally we can conclude that socio-economic situation of people have effect on health care utilization and factors like income, house tenure, sex and household size was effective on utilization in all models.

This research has faced some limitations such as the study did not include supply side factors such type of facility used by patients. There are quality aspects hidden in the type of facilities patients choose which in away directly affects demand of health services.

## 5. Conclusion

According to mentioned results and the fact that access to medical care has effect on health services utilization, policy makers should act to increase the financial access of people. This may be done with identification of households and individuals in low income deciles, households with more than 65 or smaller than 5 years old or with chronic diseases members. According to age effect on health services usage and aging population of Iran, results of this study show more importance of attention to aged population needs in future years.

Regarding to the result, betterment in public medical care facilities could profit the poor people more than others in proportion, because the rich people have more tendencies to use private health care services.

In general, existing inequality in medical services use can be considered from two various aspects: (1) health policies of government, such as the role of health sector in decreasing inequality using the policies, including family physician that is useful for the poor population; (2) determinants that are beyond the authorities of health sector, and are linked to living standards of population and require inter sectoral collaboration.

Therefore such study will have an effect on the implementation or direction of health care reforms in Iran. It will logically notice the policy makers in private and public sector in the case of re-structuring of the re-designing and administration the interventions. Moreover such study could be useful in recognizing the feasible ways of collaboration and partnership to reinforce the whole health care system.
